# Process Evaluation of Internet-Based Cognitive Behavioral Therapy Intervention for Informal Caregivers

**DOI:** 10.3389/fmed.2021.725510

**Published:** 2021-11-12

**Authors:** Ieva Biliunaite, Evaldas Kazlauskas, Robbert Sanderman, Gerhard Andersson

**Affiliations:** ^1^Department of Behavioural Sciences and Learning, Linköping University, Linköping, Sweden; ^2^Center for Psychotraumatology, Institute of Psychology, Vilnius University, Vilnius, Lithuania; ^3^Department of Health Psychology, University Medical Centre Groningen, University of Groningen, Groningen, Netherlands; ^4^Department of Psychology, Health & Technology, University of Twente, Enschede, Netherlands; ^5^Department of Biomedical and Clinical Sciences, Linköping University, Linköping, Sweden; ^6^Department of Clinical Neuroscience, Karolinska Institute, Stockholm, Sweden

**Keywords:** process evaluation, ICBT, informal caregivers, feasibility, focus group

## Abstract

**Background:** Informal caregivers are individuals who provide care for ill, frail, or otherwise dependent family members, siblings, or friends. Due to the caregiving demands, informal caregivers are known to experience negative mental health symptoms, such as stress or anxiety. Interventions based on Internet-based Cognitive Behavioral Therapy (ICBT) principles have been previously found to be effective for different populations and could also be considered as a plausible support option for informal caregivers. However, findings regarding effectiveness alone might not be sufficient for informing about the overall feasibility of the intervention.

**Objective:** The aim of this process evaluation study was to evaluate the feasibility of a previously developed ICBT intervention for informal caregivers in Lithuania. More specifically, we evaluated the suitability of the intervention in relation to its content and delivery mode.

**Methods:** Two studies were conducted. Study 1 consisted of participant evaluations of an 8-week, 8-module long therapist supported ICBT intervention. Evaluations for the Study 1 were retrieved from previously unused data, obtained from pilot testing of the intervention in which 63 informal caregivers took part. The evaluations contained of qualitative data (participant comments), as well as quantitative data (evaluations of each of the sessions). The Study 2 was an online stakeholder focus-group discussion conducted via Zoom. Eight stakeholders took part in the discussion, among whom there were social workers, medical professionals as well as individuals with caregiving experience themselves. Data were analyzed using descriptive statistics, thematic analysis, and data coding.

**Results:** Results of the Study 1 showed that most of the pilot randomized controlled trial participants evaluated content and format of the intervention positively. These results were complemented by the findings in the Study 2, in which stakeholders evaluated the intervention as suitable and promising. In addition, stakeholders made certain suggestions for improving the intervention's usability for the informal caregivers. This included improving the instructions, providing with more guidance, and considering personalization options.

**Conclusion:** The process evaluation helped to evaluate the feasibility of the ICBT intervention for informal caregivers in Lithuania from the two perspectives: users and stakeholders. Our findings suggest that the intervention is suitable for the target population.

## Introduction

Informal caregivers are individuals who provide care for family members, siblings, or close acquaintances who due to the chronic illness, frailty, or other reasons are not able to live fully independently. Informal caregiver involvement varies greatly from helping with general hygiene, medication intake, and up to 24 h per day support (1). Because of caregiving, many caregivers experience reduced well-being (2). Consequently, much of research efforts have focused on developing and testing possible support interventions for this population. Over the last decades, eHealth or internet interventions have been proposed as an alternative to traditional, face-to-face options. One of the benefits of internet interventions is that it can reach caregivers in remote geographical locations (3). It also provides an opportunity to reduce the treatment vs. demand gap (4), offers an alternative solution for individuals concerned with mental health stigma (5), and provides flexibility in accessing the material (6).

There are examples of internet interventions for informal caregivers. The focus has been on psychoeducation (7), information provision (8), and peer support (9). It is common to include multiple components in the interventions, such as education coupled with professional support (10). When it comes to the efficacy, the results have been described as promising (11). For example, multicomponent interventions have the potential to reduce symptoms of depression, anxiety, stress, and distress (12). Nevertheless, it is difficult to draw any firm conclusions about the existing interventions. As outlined by the Sherifali et al. (12), one of the reasons for this is the high heterogeneity of the interventions targeting various outcomes which are in turn assessed with different measures. In addition, findings of their meta-analysis found several included intervention trials to suffer from methodological limitations and hence be at high risk of bias in areas such as incomplete outcome data and blinding of participants among the other. This, therefore, leads to the conclusion that further, high quality research trials investigating internet intervention suitability for the informal caregivers are needed.

Due to its effectiveness in treating various psychiatric and somatic conditions (13) internet-delivered cognitive behavioral therapy (ICBT) could be outlined as a potentially beneficial way of psychological support for informal caregivers. Even though it is not uncommon for existing interventions to include certain Cognitive Behavioral Therapy (CBT) components, to the best of our knowledge, there were very few previous attempts to implement ICBT interventions for the informal caregivers. To give an example, in a recent review (14) three internet-based interventions including CBT components and targeted for dementia caregivers were reviewed (15–17). Despite including CBT components, these interventions differed in their approach regarding the guidance (guided vs. unguided) and delivery mode (computerized vs. computerized and bibliotherapy). One other example is the study by the Meichsner et al. (18) in which an existing CBT intervention was translated into the ICBT format. In this study informal caregivers were found to generally be very satisfied with the ICBT intervention which indicates its potential for this population. These findings, in combination with existing knowledge about the effectiveness of the CBT interventions, encourages further development and evaluation of the ICBT's suitability for informal caregivers.

Various frameworks exist for guiding the development, evaluation, and implementation of the internet interventions. The Medical Research Council (MRC) framework for complex interventions (interventions including several interacting components among other characteristics) (19–21) is a well-known and cited framework. It suggests that the intervention research consists of four phases: identification or development of an intervention, feasibility, evaluation, and implementation (21). There is also a set of common core elements outlined, relevant for all phases: consideration of the context, refinement of the theory, engagement of stakeholders, identification of uncertainties, refinement of the intervention and consideration of the economic factors. The phases do not necessarily follow a linear sequence meaning, that the intervention development, evaluation, and implementation process might require one to repeat some processes or move in between the phases back or forward (20). MRC framework also draws attention to the process evaluation of trials. Process evaluation has been defined as a process of exploring various aspects within research trials, such as receipt, setting, implementation and meaning of the results involving both, quantitative and qualitative methods (22). In their most recent update (21) MRC has reiterated that methods, such as process evaluations, can help researchers to move beyond evaluating the effectiveness of the interventions and answer other relevant questions, such as why the intervention does or does not work or how it could be optimized further.

When should a process evaluation be done? To start with, it could be useful following the development on an intervention, as the obtained knowledge could then be applied for investigating quality, feasibility, and prospects for implementation. At the same time, process evaluations could be conducted in other phases of development. For example, it could be useful after pilot testing of efficacy, to either help in interpreting the results or, to provide with additional evidence (23). Moreover, process evaluations could be conducted several times, at different stages of the intervention's development and analysis processes, for monitoring the quality and the treatment throughout the development process. In terms of evaluation of fidelity, it is advisable to involve stakeholders, such as users, health professionals, or other relevant stakeholders (24). Consequently, depending on the process evaluation's aims, researchers can choose to collect either quantitative or qualitative data, or do both (22).

In this paper, we present the results of a process evaluation of an ICBT intervention for reducing informal caregiver burden. We recently conducted a pilot randomized controlled trial (RCT) (25) and a qualitative study (26) for evaluating the efficacy and acceptability of this intervention for Lithuanian informal caregivers. The ICBT intervention was an eight-week long, therapist-supported program aimed at adult informal caregivers. Following the pilot RCT with 63 informal caregivers we found that the intervention reduced caregiver burden as well as depression and anxiety symptoms (between group Cohen's d = −0.70, −0.69 and −0.74 respectively). Further, the intervention resulted in reductions of stress and improved quality of life (Cohen's d = −1.06 for stress and 0.8 and 0.85 for quality of life). The results of the qualitative study showed that the informal caregivers accepted the intervention, with its format and the contents overall were valued positively (26). At the same time some differing opinions were observed in relation to the therapist-support, content, and the format of the intervention with some participants preferring it more and others less. Based on the findings of these two studies we have concluded, that even though results are promising, some information is still lacking. Firstly, content evaluation data retrieved during the pilot RCT trial needed to be utilized to further evaluate interventions suitability in the light of the some of the controversies outlined following the qualitative study. Secondly, our findings were solely based on the participant experiences. Evaluation by health professionals, social workers, or other relevant parties was needed to evaluate sustainability and implementation of the intervention. Therefore, we decided to run an evaluation phase and further investigate feasibility before planning a larger RCT. For this purpose, process evaluation was deemed as the most appropriate approach for utilizing previously collected, but not used data in combination with data representing stakeholder perspectives.

The main objective of the present paper was to conduct a process evaluation study for examining feasibility of the ICBT intervention for reducing informal caregiver burden. More specifically, we focused on evaluating delivery, content, and suitability for the target population. For this purpose, two research studies were conducted and will further be presented separately. The findings in this study will hopefully guide further development and implementation of the intervention.

## Methods

**Figure 1:**
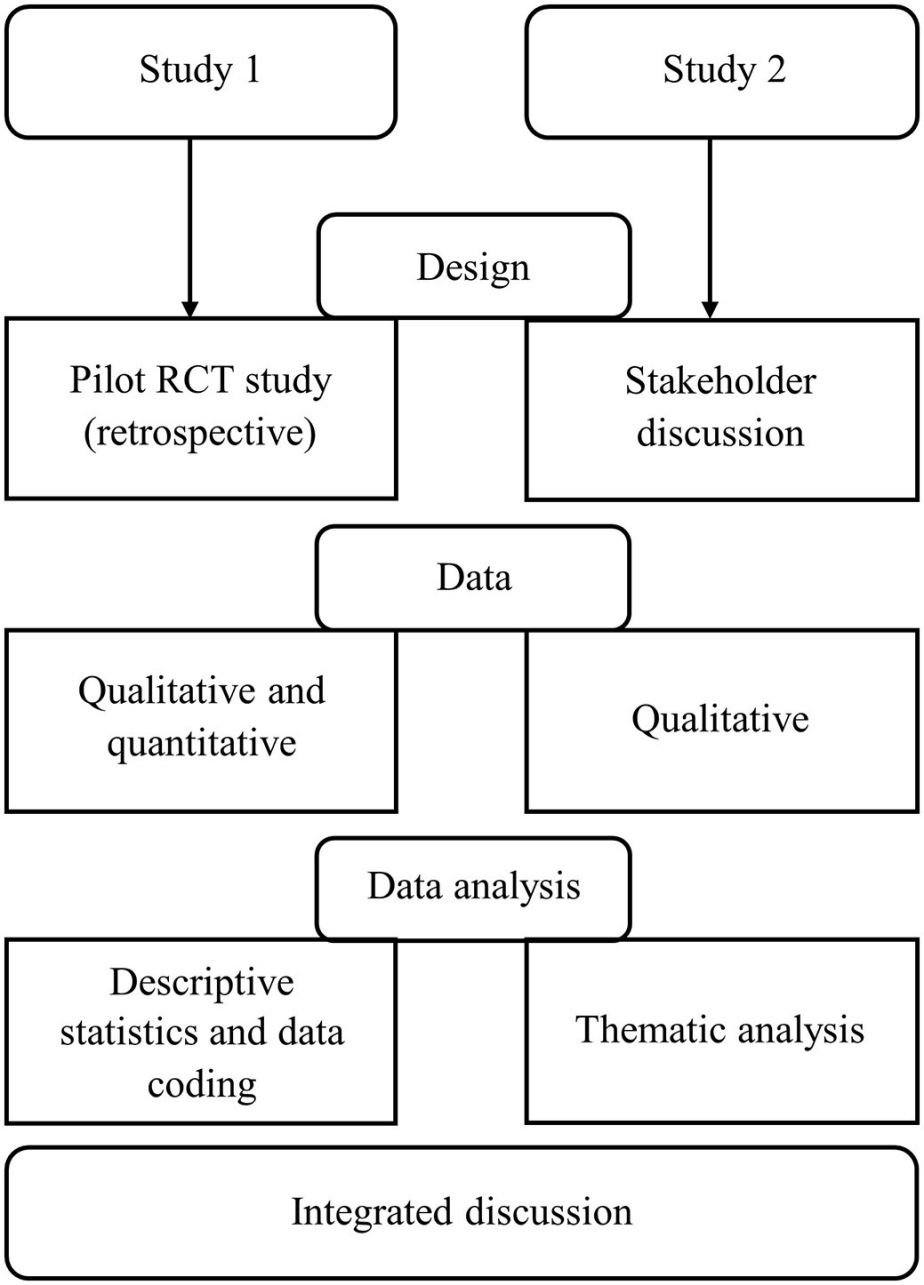
Process flow of the studies procedures.

This process evaluation study was performed following the efficacy and intervention acceptability studies. The study design was driven by recommendations provided by Moore et al. (23). To meet our goal, two separate research studies were conducted (Figure 1). The Study 1 was based on retrospective, but previously not used participant data collected during pilot RCT study (25). Study 2 was a stakeholder focus group discussion. We start by describing the intervention's development process. Representation of the two studies follows further.

### Development of the Intervention

The intervention was developed in three main steps: (1) concept development and considerations, (2) selection of the content, (3) revision of the content by a Lithuanian research group of clinical psychologists for cultural appropriateness and relevance.

#### Concept Development and Considerations

The idea to develop a guided internet-delivered self-help intervention for informal caregivers in Lithuania was initiated in association with an EU-project (ENTWINE) and was a collaboration between Linköping University in Sweden, Vilnius University in Lithuania, and the University of Groningen in the Netherlands. Few most important aspects were agreed upon on this stage. Firstly, the Iterapy online treatment platform (27) was chosen for running the intervention. Secondly, it was decided that the intervention should be therapist-guided as such interventions were found to be more effective and better adhered to than non-supported ones (28). Lastly, it was decided that the intervention should be transdiagnostic. That is, to cover several topics that would suit a broad range of informal caregivers. The main motivation to choose this type of focus was that transdiagnostic interventions account for comorbidity, which is common in mental health disorders (29).

The intervention was named ‘Slaugau artima’ which translated from Lithuanian language means I take care of my close one. In the Lithuanian context this was decided to be the closest as well as culturally understandable approximation of the English term informal caregivers.

#### Selection of the Treatment Content

A CBT approach was chosen as theoretical framework. This decision was prompted by accumulating evidence in support of ICBT for adults experiencing psychiatric and somatic conditions (6). Following this, the literature was consulted for obtaining knowledge about the most faced challenges and psychological health outcomes for caregivers. Eight main themes were selected, resulting in eight treatment modules: Introduction, Thoughts, Stress and relaxation, Problem solving, Communication, Anxiety, Behavioral activation, and Maintenance. Content for the themes was then retrieved from existing ICBT intervention programs on anxiety and mood disorders at Linköping University. Detailed description of the content of the intervention is provided elsewhere (25).

The selected content was put together for each of the topics and then translated into Lithuanian language by Lithuanian speaking research group members. Initial checks for the comprehensiveness and appropriateness of the content for the target population were then conducted. Since the intervention targeted Lithuanian informal caregivers, much attention was dedicated for making the content culturally appropriate. From the initial stage throughout the development of the intervention, adjustments of intervention content to local cultural context were discussed in the Swedish and Lithuanian research teams. Also, a small convenience sample of stakeholders from the researchers' network were consulted when necessary.

#### Revision of the Content

**Figure 2:**
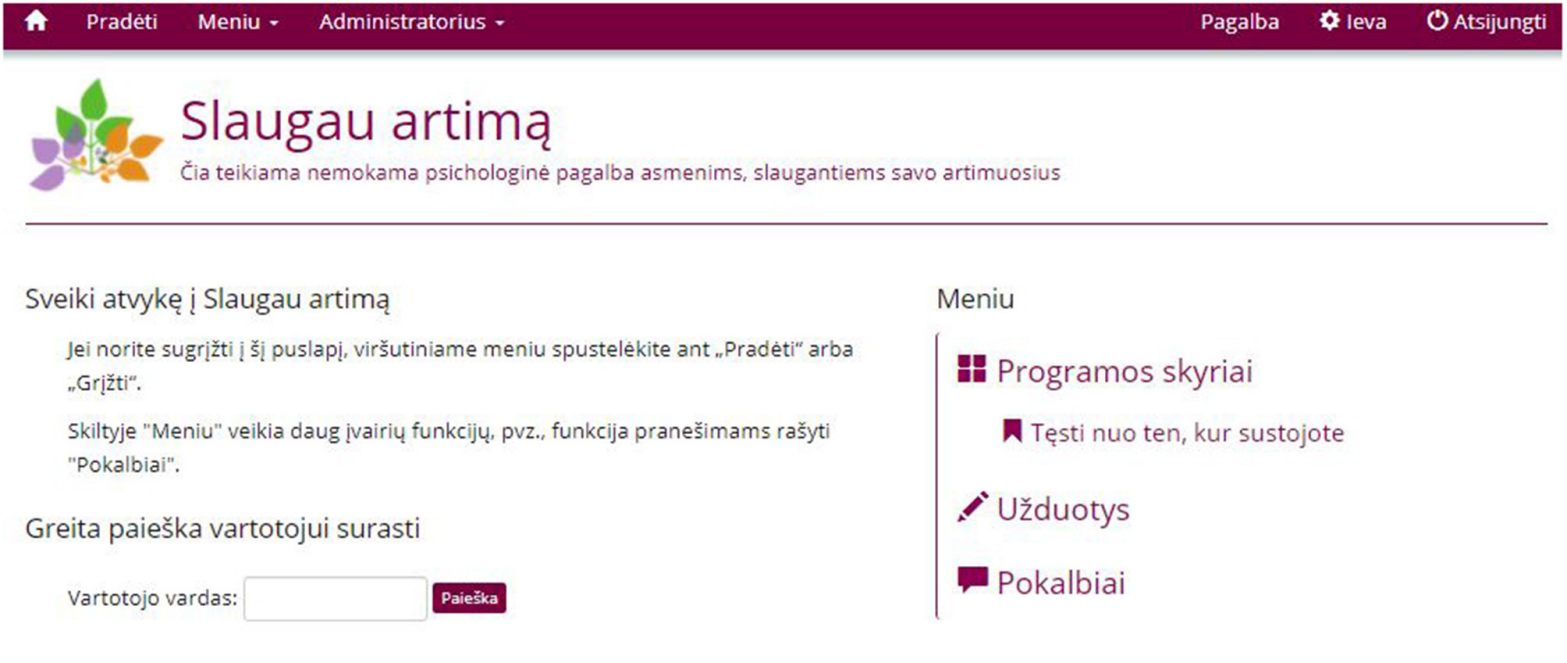
User screen after logging in. On the right-side of the screen users can click on *the Programos skyriai* to access different modules, click on *Užduotys* to access exercises or start a conversation with a psycholigst by clicking on *Pokalbiai*.

**Figure 3:**
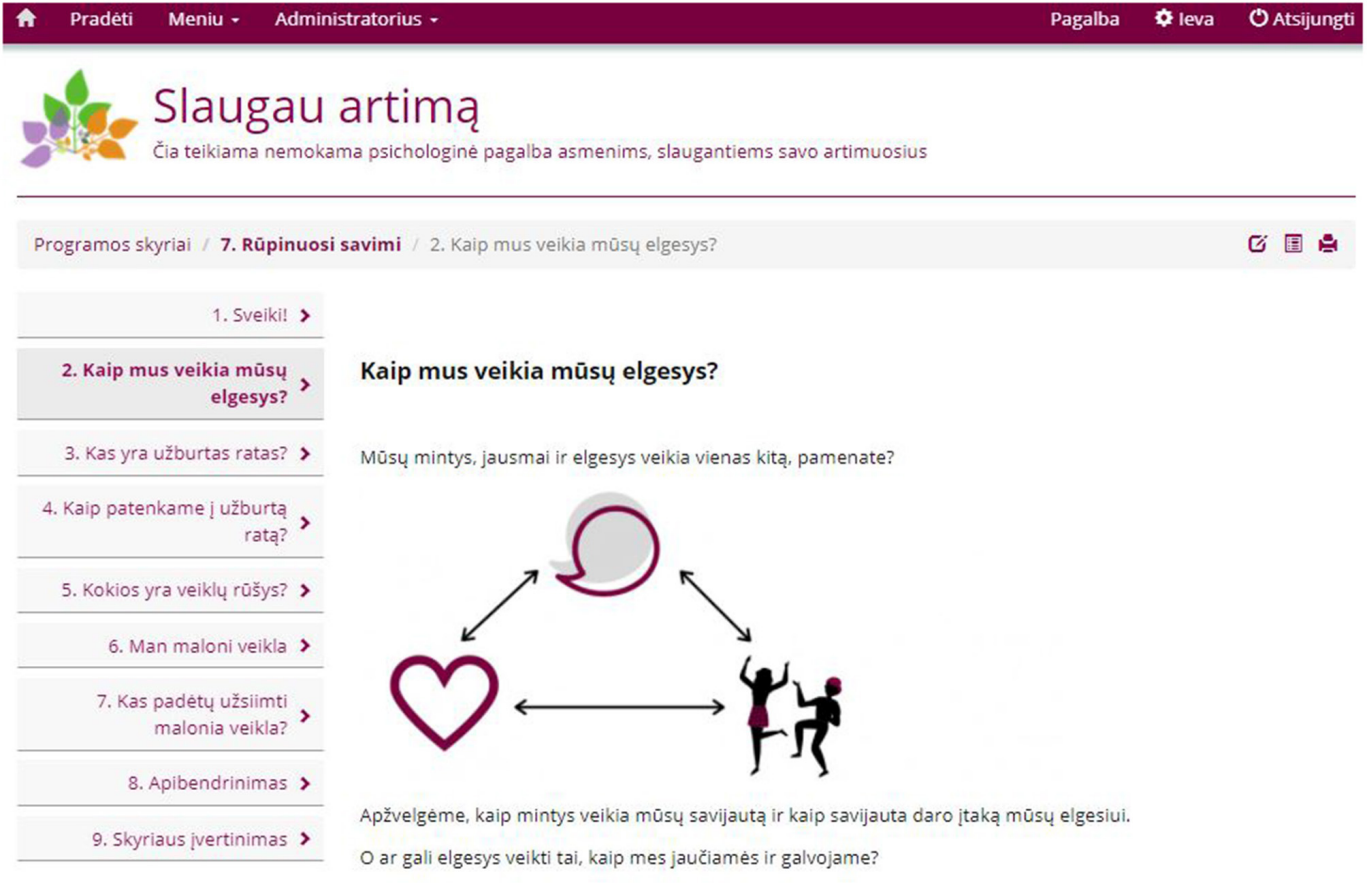
Sample of the intervention's content. On the left side, users can move from tab to tab to access modules materials (Starting with *1. Sveiki!*).

In the first stage of the revisions, a Lithuanian fluent student assistant reviewed the content of the intervention for its suitability and comprehensiveness. Certain observations were noted regarding the use of language, case examples and the structure of the content which were communicated to the research team so that changes could be made where appropriate. Following this, several discussions in the research group, including experienced clinical psychologists and researchers from Lithuania, took place. Once again, the content was revised and adapted when needed. The intervention was finalized when there were no further revisions to be made regarding the content, cultural appropriateness, and the use of language. Figure 2 represents the main page of the intervention after logging in. In the Figure 3 a sample of the intervention's content is presented.

## Study 1

### Design

In Study 1 we retrieved previously unused intervention evaluation data obtained from the participants during the pilot RCT study (25). Participation in this research study was voluntary and all participants provided informed consent prior the start of the trial. Ethics approval for the study was granted by the Vilnius University Psychology Research Ethics Committee documented as 08-07-2019 No.26. No monetary compensation was provided.

### Participants

A total of 63 informal caregivers were included in the pilot RCT. Most of the participants were female (90%) with an average age of 52 years (SD = 8.4). Participants displayed high burden (M = 53.92, SD = 12.66) as measured by the Caregiver Burden Inventory (30). A complete list of the characteristics is provided in the Supplementary Material.

### Materials

Data retrieved from the pilot RCT participants consisted of module evaluation questions as well as an additional question about the content that participants were provided at the start of each module. Module evaluation questions consisted of four questions and one open box answer (Table 1). For this study, we did not incorporate the first question asking about the well-being of the participant. In addition, at the start of each week's module (session) participants were able to share their thoughts, ideas, or experiences in relation to the previous weeks content or their well-being (Table 1).

**Table 1:** List of module evaluation questions for the pilot RCT participants.

**Question**	**Rating**
**I Module evaluation questions**	
What can your rate your current well-being?	1-very bad; 5-very good
Approximately what percentage of all the given information did you read?	0–100%
How do you overall rate this session?	1-very bad; 5-very good
How much time did you spend for reading the materials and conducting the exercises?	1 min−2 h
Here you can type in any thoughts, feelings, or insights that you have experienced during this session.	Open text answer
**II Question at the start of the module**	
Last week we have learnt about (), did you manage or have you tried to apply ()?^*^ If you would like to share your feelings, thoughts, or experiences following (…) session, you can do so in the box below.	Open text answer

* *At the start of the each week's module (session) participants were usually shortly reminded about the previous weeks topic and then shortly requested if they tried or managed to apply gained knowledge*.

### Procedure

The pilot RCT study was conducted between October 2019 and March 2020. Participants in the study were able to choose to evaluate each of the eight modules as well as to share their experiences of applying the knowledge at the start of the each of the new modules. Answering intervention evaluation questions was on a voluntary basis and took only a few minutes.

### Analysis

Quantitative data were analyzed using IBM SPSS Statistics (version 25). Descriptive statistics were used for summarizing quantitative responses. The qualitative data were coded after reading all the text responses provided by the participants (31). First, participant comments were open coded. Then, codes were reviewed, scrutinized, and integrated into categories.

### Results

#### Quantitative Findings

Participant responses to multiple choice module evaluation questions are summarized in Table 2. The number of responses to module evaluation questions gradually decreased throughout the duration of the intervention: from 45 out of 63 (71.4%) at the start (Module 1) to 26 out of 63 (41.3%) at the end (Module 8). As the findings presented in the Table 2 illustrate, the majority of the participants have read all the of the given information for each of the modules (from 77.1 to 93.5%). Consequently, the majority rated modules as good (24.3–46.2%) or very good (33.3–57.7%) with exception of module 5 (Communication), which was mostly rated as very good (51.4%) or neither good nor bad (31.4%). Lastly, most of participants spent between 30 min and 1 h for engaging with each week's material (35.6%-60%) except for Module 1 (51.1% spent 1–30 min).

**Table 2:** Summary of the multiple-choice module evaluation answers from pilot RCT participants.

	**List of modules**							
	**First**	**Second**	**Third**	**Fourth**	**Fifth**	**Sixth**	**Seventh**	**Eight**
**Total responses**	45	39	38	38	35	37	31	26
**Read info** ***n*** **(%)**								
50%	1 (2.2)	0	0	1 (2.6)	0	0	0	1 (3.8)
75%	7 (15.6)	4 (10.3)	6 (15.8)	7 (18.4)	8 (22.9)	5 (13.5)	2 (6.5)	1 (3.8)
100%	37 (82.2)	35 (89.7)	32 (84.2)	30 (79.0)	27 (77.1)	32 (86.5)	29 (93.5)	24 (92.4)
**Ratings** ***n*** **(%)**								
Very bad	0	0	0	0	0	0	0	0
Bad	2 (4.4)	2 (5.1)	0	1 (2.6)	1 (2.9)	1 (2.7)	0	0
Neither good nor bad	11 (24.4)	6 (15.4)	6 (15.8)	8 (21.1)	11 (31.4)	7 (18.9)	9 (29.0)	2 (7.7)
Good	15 (33.4)	18 (46.2)	12 (31.6)	11 (28.9)	5 (14.3)	9 (24.3)	10 (32.3)	9 (34.6)
Very good	17 (37.8)	13 (33.3)	20 (52.6)	18 (47.4)	18 (51.4)	20 (54.1)	12 (38.7)	15 (57.7)
**Time spent** ***n*** **(%)**								
1–30 min	23 (51.1)	9 (23.1)	5 (13.2)	6 (15.8)	4 (11.4)	8 (21.6)	7 (22.6)	8 (30.8)
30 min−1 h	16 (35.6)	19 (48.7)	21 (55.2)	21 (55.3)	21 (60.0)	20 (54.1)	17 (54.8)	13 (50.0)
1–2 h	5 (11.1)	9 (23.1)	7 (18.4)	7 (18.4)	7 (20.0)	7 (18.9)	5 (16.1)	3 (11.5)
2>h	1 (2.2)	2 (5.1)	5 (13.2)	4(10.5)	3 (8.6)	2 (5.4)	2 (6.5)	2 (7.7)

*List of modules: (1) Introduction, (2) Thoughts, (3) Stress and relaxation, (4) Problem solving, (5) Communication, (6) Anxiety, (7) Behavioral activation and (8) Maintenance*.

#### Qualitative Findings

A summary of the results is presented in Table 3. A total of 323 comments were retrieved. These were either content specific experiences (*n* = 176) or participant reflections (*n* = 147). Latter comments were not coded further, as it represented individual's personal experiences and thoughts. Content specific comments were divided into three main categories: Content/format positive (72.2%), Content/format hesitant (20.4%) and Content/format negative (7.4%). Short definitions and examples of participant comments illustrating each of the categories and sub-categories are presented in Table 4.

**Table 3:** Summary of the open-ended module evaluation answers from pilot RCT participants.

**Categories**	**Overall (*n* = 323)**	**At the end of module^a^ (*n* = 196)**	**At the start of module^b^ (*n* = 127)**
I Reflections	147 (45.5%)	90 (45.9%)	57 (44.9%)
II Content specific comments	176 (54.5%)	106 (54.1%)	70 (55.1%)
▪ Content/format positive	127 (72.2%)	71 (67.0%)	56 (80.0%)
▪ Learning about thoughts	40 (31.5%)	20 (28.2%)	20 (35.7%)
▪ Overall applicability	30 (23.6%)	21 (29.6%)	9 (16.1%)
▪ Being able to relax	17 (13.4%)	12 (16.9%)	5 (8.9%)
▪ Dedicating time for own needs	16 (12.6%)	4 (5.6%)	12 (21.4%)
▪ Other (e.g., problem solving)	24 (18.9%)	14 (19.7%)	10 (17.9%)
Content/format hesitant	36 (20.4%)	27 (25.5%)	9 (12.9%)
Content/format negative	13 (7.4%)	8 (7.5%)	5 (7.1%)

a *Question at the end of the module: Here you can type in any thoughts, feelings, or insights that you have experienced during this session*.

b *Question at the start of the module (requesting about previous week's content): If you would like to share your feelings, thoughts, or experiences following (…) session, you can do so in the box below*.

**Table 4:** Examples of participant comments categorized into Content specific category.

**Category**	**Definition**	**Examples**
Content /format positive	Comments representing participant appreciation of the intervention's content or format.	
Learning about thoughts	Benefit of better understanding thought processes.	*Alternative thoughts helps me to have a more positive outlook towards the future*. *Less chaos in thoughts and feelings*.
Overall applicability	Ability to apply intervention's content and *suitability of the format*.	*I have applied a lot of things*. *For me it was really useful; I was able to get to know myself better as well as my own feelings and disappointments*.
Being able to relax	Ability to relax and applicability of relaxation exercises.	*Provided meditation methods put me in a good mood*. *Exercises enable (me) to relax a little bit*.
Dedicating time for own needs	Learning about benefits of spending time for oneself.	*It is necessary to pay more attention to oneself*. *I am starting to manage to find time for myself and engage in pleasurable activities*.
Other	Comments in relation to problem solving, improving communication quality or sharing with the therapists	*I liked compiling a list of problem-solving solutions*. *It is easier for me to communicate with mum*. *I don‘t feel alone*.
Content/format hesitant	Comments representing participant uncertainty about the intervention's content, format, applicability of the information or their own ability to apply it.	*I have experienced a lack of faith in the usefulness of the exercises; or perhaps (the lack) of will conduct them*. *It is difficult to express thoughts via writing*.
Content/format negative	Comments representing participant dislike of the intervention‘s content or its applicability.	*The topic of this session was completely not in accordance with my situation*. *Taking part in this program has started to irritate me as it requires additional time*.

Comments in the first, Content/format positive category represented aspects of the intervention that the participants appreciated. These comments were further sub-categorized into five smaller groups. The two biggest ones were Learning about thoughts (31.5%) and Overall applicability (23.6%). Comments in the Learning about thoughts sub-category indicated participants to benefit from the knowledge about their own thought patterns. Also, to benefit from information about how to interpret their thoughts and how the negative automatic thoughts could be changed into the less-negative alternative ones. Consequently, participants comments in the Overall applicability sub-category indicated participants to be generally able to select and apply the provided information. Also, these comments indicated participants to find the content as well as the format of the intervention overall acceptable and suitable. The remaining three sub-categories in the Content/format positive category were Being able to relax (13.4%), Dedicating time for own needs (12.6%) and other (18.9%). Firstly, the comments in the first two sub-categories indicated participants to benefit from the relaxation techniques as well as the parts of the content, that encouraged to focus on own needs. By some, intervention itself was perceived as a means of distraction from daily routine and an opportunity to spend the time for one-self. In turn, the comments in the sub-category other were either in relation to learning to problem solve (5.5%), improving quality of communication with the close ones (5.5%), or ability to share own thoughts and experiences with the therapist (7.9%). That is, to very specific components of the intervention, namely, the problem solving and communication skill related content as well as the function allowing to communicate with the therapist.

The remaining two categories were the Content/format hesitant (20.4%) and Content/format negative (7.4%). It could be summarized, that the main difference between these two categories was that participant comments in the latter category expressed more direct dislike or lack of approval as opposed to hesitancy or reluctancy toward engaging with the intervention. To start with, comments in the Content/format hesitant category reflected participant reservations about either the content and format of the intervention or own abilities in applying the intervention's materials. That is, some of the comments expressed participant doubts regarding content's applicability to their own situation, with some of the topics or exercises being perceived as less relevant for individual caregivers. In addition, some participants expressed uncertainty or doubts about how some of the exercises should be conducted or, were not certain if they are able to conduct such exercises correctly. Also, there were comments that indicated some participants to experience difficulty in translating the knowledge from the intervention into the daily life situations. In turn, as indicated by the categories title, comments in the last category, Content/format negative, represented participant negative attitude toward intervention's format or applicability of the content. Most of the comments in this category could be summarized to illustrate participant critique toward the content or exercises as being not suited for their needs or circumstances. In addition, comments in this category also indicated some of the caregivers to be longing for more intensive contact with the therapists. Lastly, some comments indicated participants to be burdened with the fact that the intervention required time to engage with.

## Study 2

### Design

Study two was set up as a stakeholder discussion. Informed consent was obtained from all participants. Ethics approval for this study was not required according to the national ethical regulations for research, as it was not a clinical trial, participants were not requested to share any sensitive information and no obvious risks could be identified. Participation in the focus group discussion was completely voluntary and no monetary compensation was offered.

### Participants

We defined stakeholders as individuals who were connected to the informal caregiving either via personal experiences or professional capacity (21). As a consequence, we contacted several organizations, such as the Huntington's disease association or association of multiple sclerosis, with invitation to take part in the focus group discussion. In addition, researchers also reached out to the existing contacts, known to either have informal caregiving experience or acquaint with informal caregivers via their professional setting. As a result, a convenience sample of eight female participants was recruited (Table 5). Participants ages ranged from 39 to 58 years, with an average age of 47.71 years (SD = 7.66) One participant did not provide personal details. All participants had professional experience in healthcare or social services and had obtained higher education diploma. Three were involved in the social work, one was a medical doctor, one was changing career from economy to psychology, one was a state employee, and one was a teacher and an informal caregiver. Participants were residing in various parts of the country. After being included participants were able to view a short presentation introducing the intervention.

**Table 5:** Focus group participant characteristics.

**Name^a^**	**Age^b^**	**Residence^c^**	**Education**	**Occupational area**
Sara	39–49	North	University degree	Social work
Iris	39–49	South-East	University degree	Transitioning
Ann	50–59	North	University degree	Social work
Rose	50–59	Middle	College degree	Social work
Mia	39–49	Middle	University degree	Medicine
Tess	50–59	West	University degree	Education
Lily	39–49	South-East	University degree	Governmental
Ida	Missing	Missing	Missing	Missing

a *The names in the table are pseudonyms*.

b *Age in years is presented in the approximate range*.

c *Area of residence in Lithuania*.

### Materials

The discussion guide included the meeting's agenda, several broadly phrased questions, and several question probes. The discussion part was structured to cover three main aspects in relation to the intervention: (1) information provided in the public pages of the intervention, (2) format and the content of the intervention and (3) the communication function between the participants and the therapists. Some of the questions posed to the participants were: What is your first impression after viewing this? What are your thoughts after seeing this? and Now that you have seen it, what is your opinion about the structure of the content?

### Procedure

In preparation for the discussion, the main author of the paper (IB) and a student assistant familiar with the intervention pilot tested the procedure using the discussion guide. The stakeholder focus group discussion took place in November 2020 online, via Zoom and was recorded following consent from all participants. IB chaired the discussion and the student assistant took the notes. The discussion started with a general introduction. Before recording, participants were once again asked for verbal consent. All three parts of the intervention relevant for the discussion (publicly available information, format and the content and communication function) were introduced and discussed separately. Following the focus group discussion, participants were provided with a short summary of the main discussion points. Participants were also encouraged to contact the researchers with suggestions or corrections regarding the discussion's summary.

### Qualitative Data Analysis

Data analysis was performed using inductive reflexive Thematic Analysis (TA) (32, 33) conducted within realist paradigm framework. First, data were transcribed. After this, transcripts were manually coded. This resulted in a list of initial codes. These, in turn, where then collated to form themes. Themes were reviewed resulting in some of the codes being re-coded and re-grouped. This process was repeated until the themes were fully refined. Since our main goal was to explore stakeholder impressions and experiences, themes were identified on a semantic level, without putting too much focus on ideologies or underlying assumptions. Analysis process was conducted by the main author and the student assistant with the joint expertise of the remaining co-authors when necessary.

### Results

**Figure 4:**
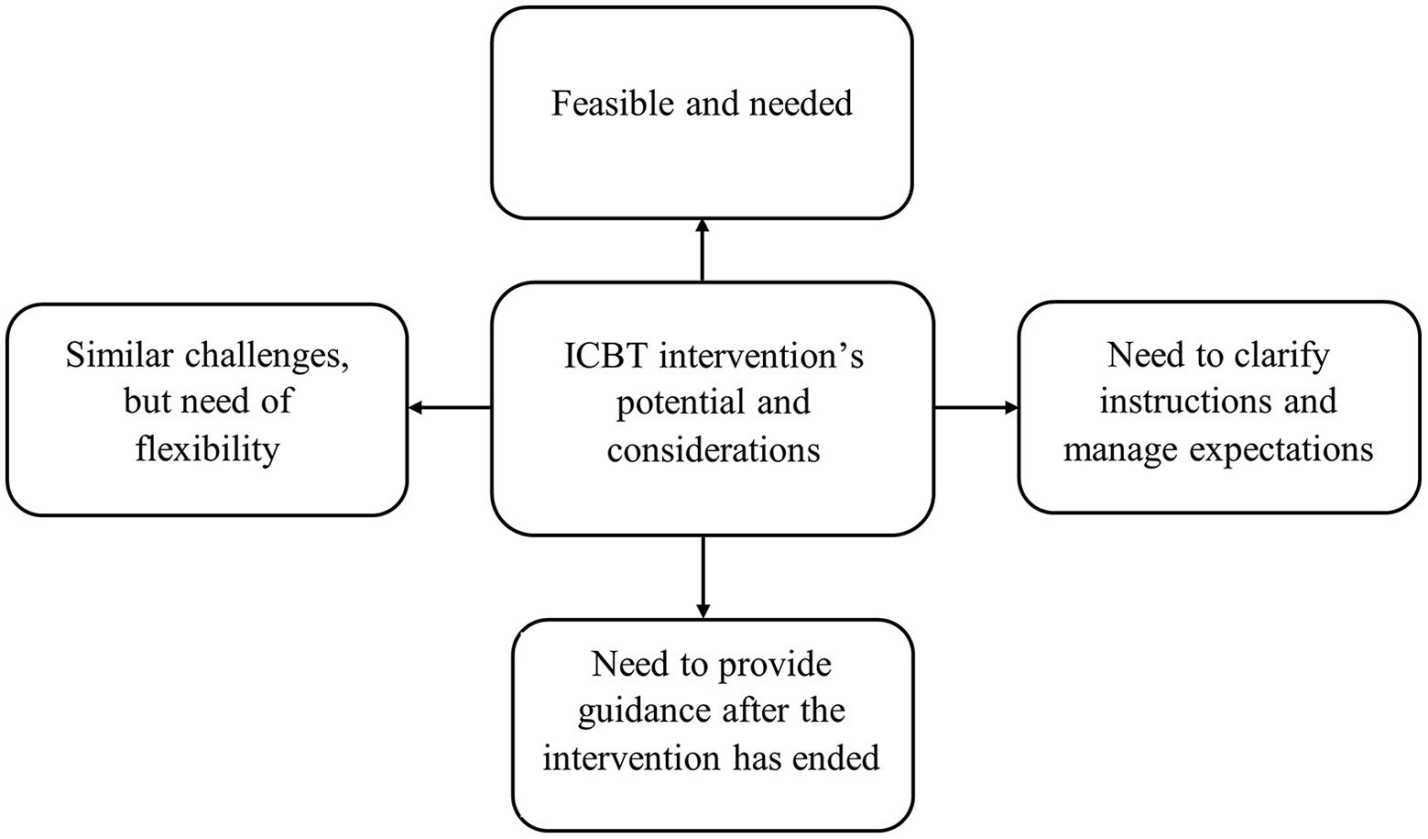
Main theme and sub-themes generated following stakeholder focus group discussion.

The focus group discussion lasted for 1 h and 42 min. Following data analysis, one main theme titled *ICBT intervention's potential and considerations* and four sub-themes were generated. The sub-themes are: (1) feasible and needed, (2) need to clarify instructions and manage expectations, (3) need to provide with guidance after the intervention has ended, and (4) similar challenges, but need of flexibility. The first sub-theme (Feasible and needed) illustrates benefits of the current ICBT intervention. The remaining three present several suggestions that could be implemented for maximizing intervention's benefits for the caregivers. Illustration of the themes and sub-themes is presented in the Figure 4. Description of the findings is provided in the text as well as quotes in Table 6.

**Table 6:** Selected examples of focus group participant quotes.

**Theme and sub-themes**	**Quote examples**
▪ ICBT intervention's potential and considerations	
▪ Feasible and needed	*Everything is listed clearly, information is also comprehensible, structured*. *For the beginning, it's a very good platform, good basis and, I think, a good starting point*.
▪ Need to clarify instructions and manage expectations	*A person might even get a little frightened not knowing how much time (participation) will require*. *This is just like for everything: it is important that they (caregivers) know*.
▪ Need to provide guidance after the intervention has ended	*The continuation (of support) would be really necessary*. *In my opinion, such contacts would also be of a great help for these people*.
▪ Similar challenges, but need of flexibility	*For others it might be the other way round, there should be some kind of option to choose*. *The more options, the higher number of happy clients*.

Discussion regarding the intervention's content was accompanied by the view, that informal caregivers, despite their unique circumstances, are often faced with similar psychological challenges. As described in the first sub-theme Feasible and needed, stakeholders overall expressed a positive attitude toward the intervention. More specifically, the intervention's format, selection of themes and ability to contact the therapists were appreciated. On the other hand, stakeholders emphasized that despite being faced with similar demands, each informal caregiver has a unique set of personal characteristics, has different caregiving experience, and is faced with unique challenges. Hence, the main overarching idea behind the remaining of the three sub-themes is that current version of the intervention could be developed further to offer valuable support for many, despite their different circumstances. That is, by providing options for personalization and by offering clearer instructions as well as options for the continuity of the support.

#### Feasible and Needed

Stakeholders reflected on how much psychological support is needed as well as the lack of resources for informal caregivers in Lithuania. The intervention was seen as an accessible and suitable support option that could bridge this existing gap. In addition, stakeholders appreciated that the intervention covered a range of different topics relevant for informal caregivers. Also, that the intervention was seen as well-structured.

*One can see that a lot of work has been put into this; a lot of materials, systemised; included (information) is really useful and needed. (Ida)*

One other outlined aspect was the communication between the informal caregivers and therapists. Stakeholders positively reflected on the fact that informal caregivers were able to communicate with their assigned therapist throughout the duration of the intervention. This function was also found to be useful for encouraging caregivers to engage with the materials. Also, such function was deemed important for allowing them to share their personal experiences and challenges. Lastly, the fact that the intervention is delivered online was also perceived positively. This finding is especially valuable considering the ongoing COVID-19 pandemic at the time of data collection and the reduction in availability of face-to-face support.

*At this moment, such support is more relevant than live support. (Ann)*

#### Need to Clarify Instructions and Manage Expectations

In addition to recommending including options for personalization stakeholders reflected upon certain aspects that could improve prior expectations and the ease of using the intervention. To start with, two main aspects were outlined by the stakeholders regarding prospective user expectation management: time involvement and relationship with the therapist. Stakeholders emphasized the importance of informing informal caregivers about how much time would be required for engaging with the intervention. This was mentioned because caregivers often have a limited amount of time to spare. Including at least an approximate estimation of time could prepare them for scheduling the time around their duties. In addition, it was suggested that more information should be provided regarding the frequency of communication with the therapist. That is, how soon the participant will receive a response, how frequently communication will take place and what format it will take.

*The more there is clarity, the easier everything is, the less questions arise. (Ann)*

In terms of the ease of using the intervention, two main points were outlined. First, stakeholders suggested that more quick access options should be included in the intervention. That is, to provide certain shortcuts and a clear sequencing for accessing different topics and exercises. This was not only to make it more user friendly, but to also to save time for the caregivers. Second, stakeholders discussed including additional guidelines about using the intervention and especially, the technical parts of it, such as logging in, since not all the informal caregivers might be equally confident or affinitive in using online interventions.

…*some instructions would help at least a bit for some. (Rose)*

#### Need to Provide Guidance After the Intervention Has Ended

This subtheme reflects the need for continuation of support. Stakeholders discussed the importance of providing participants with different information sources about where one could reach out for support once the intervention had finished. For this, several suggestions were made. For example, to provide a list of helplines that offer mental health support, to provide with links to literature, useful web resources and possibly even online support communities. Such information regarding further support was stressed as an additional and essential tool for informal caregivers in allowing them to maintain their well-being and further apply the knowledge gained throughout the course of the intervention.

*So not to leave (them) (…) in the hands of the fate, so that they could further reach out somewhere else in the future. (Ida)*

#### Similar Challenges, but Need of Flexibility

Even though stakeholders described the topics of the intervention as useful, it was also commonly agreed upon that to reach and benefit informal caregivers in their differing circumstances, certain personalization options could be implemented. For example, to give participants an opportunity to choose the topics they want to start with instead of providing everyone with the same sequence of the topics.

*My idea was that (…) a person could switch things around based on what theme is the most relevant for him at the moment. (Lily)*

Other aspects that were mentioned were to include a wider range of selection options for either listening to information, or viewing it, since majority of the intervention's content is currently provided in text. Lastly, the opportunity for tailoring the communication with the therapist function was also discussed. Stakeholders suggested that due to individual differences, some of the participants might benefit for an opportunity to call or reach the therapist in other ways in addition or instead of currently implemented messaging function.

…*hybrid way is good (…) meaning that it is possible to mix-it up, really. (Iris)*

## Discussion

In this paper we aimed to conduct a process evaluation investigating the feasibility of ICBT intervention for informal caregivers in Lithuania. More specifically, we have aimed to evaluate the intervention by examining its delivery, content, and suitability for the target population. Two studies were conducted. In Study 1 we have analyzed evaluation data obtained from the informal caregivers who took part in a pilot RCT study for efficacy of the intervention (25). Study 2 was an online focus group discussion with eight stakeholders. Following data analysis, several aspects regarding intervention's feasibility were outlined. We further discuss these findings jointly.

### Intervention's Feasibility

#### Feasibility of the Content

Most of the participants who filled in the module evaluations sheets spent between 30 min and 1 h for engaging with the module's materials, read all the provided information and rated modules as mostly good or very good. The latter is further evidenced by the finding that a majority of the coded pilot trial participant comments fell into the *Content/format positive* category. *Learning about thoughts (*the CBT explanation of relation between thought, emotions, and behavior) and *Overall applicability* were the two largest sub-categories in this group. This is in accordance with the *Feasible and needed* theme generated following focus group discussion in Study 2. Stakeholders found the intervention useful due to its clear structure and coverage of a range of topics. Clear structure, comprehensiveness and helpfulness of the content was previously outlined as beneficial in other ICBT studies (34). The opportunity to learn about relaxation methods, obtain knowledge about problem solving and communication were also found to be appreciated by the informal caregivers. As can be seen from the *Dedicating time for own needs* sub-category, the intervention also encouraged caregivers to focus on themselves. It is evident, that due to the caregiving demands and other responsibilities caregivers must often put their needs aside (1). As our findings illustrate, the intervention can help to bring this focus back.

#### Communication With the Therapist

Stakeholders viewed therapist support as beneficial for the caregivers. Specifically, for supporting them throughout the intervention period and allowing to share their experiences. On the other hand, only a fraction of the caregiver comments fell into this category (7.9% of the comments in *Content/format positive* category). It could be stated that the stakeholders were more expressive about the benefits of such support. As it is evident from our previous qualitative work in evaluating the acceptability of the ICBT intervention, not all informal caregivers appreciated the therapist support equally (26). This has been observed in previous research studies, with some of the users desiring for more support and contact with the therapist (35). Despite this, since this function was supported by at least a part of informal caregivers and by all stakeholders and considering previous findings suggesting ICBT interventions to be more effective than non-supported ones (28), we deem that this function should also be maintained in the further evaluation of the intervention.

In sum, we conclude that the joint results of the two studies indicate that the intervention is feasible. However, as it will be evident from the following sections, certain considerations must be accounted for further development of the ICBT intervention.

### Further Development of the Intervention

Data from both informal caregivers and stakeholders outlined certain areas for intervention's improvement. In Study 1, comments in the *Content/format hesitant* category revealed that some of the informal caregivers experienced uncertainty about suitability of the material. Also, they questioned their own abilities to apply those materials. In turn, comments in the *Content/Format negative* category expressed informal caregiver dissatisfaction with the intervention or its components. Consequently, In Study 2, two of the themes reflected stakeholder suggestions about improving the intervention. That is: *Need to clarify instructions and manage expectations* and *Need to provide guidance after the intervention has ended*. The last theme*, Similar challenges, but need of flexibility* encourages us to think further about how to increase the flexibility of the intervention, so that it could be suitable for caregivers despite their differing circumstances. We discuss each of these points below.

#### Reducing the Hesitancy by Improving the Instructions

Not providing prospective users with enough information prior to the intervention might be one of the initial causes for participant non-engagement (36). In turn, providing clear instructions and information early on could help to build participant trust in the intervention and their confidence in using it (37). The latter could be important for informal caregivers, who might have negative prior experiences of using such interventions (38) or, consider themselves as less tech savvy (39). Including a ‘search function’ could also be another development allowing future users a quicker access for content related information as well as intervention use instructions (40). In addition to providing clear instructions, the stakeholders suggested providing information about the time needed for engaging with the intervention. Difficulty to integrate the use of the intervention into one's life due to the limited amount of time has been previously found to be a barrier for engagement with the intervention (38). Clarifying this early on could help to manage informal caregiver expectations and hence, engagement. The latter also applies for communication with the therapist. That is, informing about the type of communication (messaging) and the expected frequency. As a last point, stakeholders outlined a need to provide informal caregivers with guidance after the intervention. Even though it is out of the intervention's scope to provide with extensive list of informational resources, a list of useful websites, relevant literature, or support groups could be provided to be used for after the intervention ends.

#### Considering Informal Caregiver Differences and Similarities

The informal caregiver comments in the *Content/format negative* category represent their dissatisfaction with the intervention. Such experiences occurred either because the content or the format of the intervention did not meet their needs or did not suit their situation. Theme *Similar challenges, but need of flexibility*, generated a focus group discussion, and provides an explanation of such findings. Even though ICBT has been found to be effective in alleviating the symptoms of many psychological disorders (6), similarly as in traditional face-to-face CBT setting, effects are not equally successful for all the users. Hence, one aspect to consider is the transdiagnostic nature of the intervention. In the current version, all participants received access to the same intervention including eight themes, queued one after another. One benefit of such interventions is that they target comorbidity. Initially, a transdiagnostic approach seemed more appropriate as informal caregivers are known to experience various mental health symptoms such as for example, stress and depression (41). Also, based on the findings indicating both transdiagnostic and tailored treatments to be effective in depression and anxiety disorders (42). On the other hand, as suggested by the stakeholders, certain amount of tailoring could help to meet the needs of wider groups of caregivers. One of the solutions could be allowing caregivers to either choose the themes of the modules themselves, or request therapists to select and queue them individually, on a case-by-case basis. In such scenario, it would be possible to maintain a transdiagnostic approach and, at the same time, allow a degree of personalization.

Further development of the intervention could focus on clarifying the instructions for using the intervention, adding certain shortcuts to improve accessibility of the materials, and provide information on how to handle the situation when the intervention ends. In addition, implementation of personalization options, such as selecting and queuing themes based on each caregiver's needs, should be considered.

### Limitations

Several limitations will be discussed. To start with, module evaluations in Study 1 were not filled in by all informal caregivers who took part in pilot RCT trial. For this reason, the collected data might not accurately represent all informal caregiver experiences. Similarly, a convenience sample of eight stakeholders in the Study 2 is relatively small and might not be representative. A more comprehensive recruitment approach as well as higher sample size could have resulted in more representative findings. At the same time, stakeholders were individuals with various educational and occupational backgrounds residing in different locations spread throughout the country. Also, focus group discussions are often based on small samples. Another limitation stems from the fact that the process evaluation could have been conducted before the piloting of the ICBT intervention. Our decision was motivated by the knowledge that the basic structure of the intervention platform as well as the basic content, was researched in tens of trials (43). Therefore, this provided us with grounds for piloting the intervention first.

### Relevance and Applicability of the Findings

First, this study describes a process evaluation of the ICBT intervention for the informal caregivers. Previously, there has only been few attempts to study the suitability of ICBT for informal caregivers, in spite of the established effects of ICBT for various psychological symptoms (13). Second, our findings provide information on how the intervention could be optimized further. We hope that this knowledge can also be beneficial for other researchers who are developing and adapting internet interventions. Third, the findings provide information about the possible future implementation and acceptability of ICBT as viewed by the stakeholders. Lastly, the study adds to the knowledge in relation to the specific cultural context for which internet intervention research still is scarce.

### General Conclusion

A process evaluation was conducted for evaluating the feasibility of an ICBT intervention for informal caregivers. Most of the participant comments indicated the interventions format and content to be perceived positively. In addition, stakeholders described the intervention as needed and acceptable means of the support for the informal caregivers. Despite this, several developments could be made before further research investigating its effectiveness is conducted. To start with, clear instructions about the use of the intervention should be provided. Prospective participants should be informed about what to expect from the intervention and, what efforts will be required from them. In addition, by the end of the intervention, a concise list of further resources should be provided. Lastly, an opportunity to tailor the intervention's themes should be considered, based on informal caregiver circumstances. Once these are implemented, further evaluation of the intervention's effectiveness in a larger randomized controlled trial is warranted.

## Data Availability Statement

The datasets presented in this article are not readily available because it is not possible to fully anonymize the dataset without impacting the primary research. Requests to access the datasets should be directed to the primary investigator Prof. Gerhard Andersson, gerhard.andersson@liu.se.

## Ethics Statement

The studies involving human participants were reviewed and approved by Study 1: Vilnius University Psychology Research Ethics Committee documented as 08-07-2019 No. 26; Study 2: Ethics approval for this study was not required according to the national ethical regulations for research, as it was not a clinical trial, participants were not requested to share any sensitive information and no obvious risks could be identified. The patients/participants provided their written informed consent to participate in this study.

## Author Contributions

IB and GA contributed to the study conception, coordination, design, and data collection and analysis. IB drafted the manuscript with input from GA. EK and RS contributed to the design of the study. All authors have contributed to revising the manuscript and approved the final version of the manuscript.

## Funding

This project has received funding from the European Union's Horizon 2020 research and innovation programme under the Marie Skłodowska-Curie grant agreement No. 814072 and is part of The European Training Network on Informal Care (ENTWINE).

## Conflict of Interest

The authors declare that the research was conducted in the absence of any commercial or financial relationships that could be construed as a potential conflict of interest.

## Publisher's Note

All claims expressed in this article are solely those of the authors and do not necessarily represent those of their affiliated organizations, or those of the publisher, the editors and the reviewers. Any product that may be evaluated in this article, or claim that may be made by its manufacturer, is not guaranteed or endorsed by the publisher.
