# Supplementary material for: Process Evaluation of Internet-Based Cognitive Behavioral Therapy Intervention for Informal Caregivers
Source: Front Med (Lausanne). 2021 Nov 12;8:725510. doi: 10.3389/fmed.2021.725510 (PMC8632733; doi:10.3389/fmed.2021.725510)
Supplement: Supplementary file 1 [file Table_1.DOCX]

Supplementary Material

**Supplementary Table**. Informal caregiver sociodemographic characteristics at baseline in the pilot RCT study^1^.

| Participant characteristics | Overall (n=63) | Intervention group (n=31) | Wait-list control group (n=32) |
| --- | --- | --- | --- |
| Age caregiver (year): M (SD) | 52 (8.4) | 54 (7.9) | 50 (8.57) |
| Age recipient (year): M (SD) | 71 (21.1) | 70 (23.13) | 72 (19.28) |
| Gender caregiver: n (% female) | 57 (90%) | 28 (90%) | 29 (91%) |
| Gender recipient: n (% female) | 44 (70%) | 20 (65%) | 24 (75%) |
| Relation receiver: n (%) |  |  |  |
| Husband/wife/partner | 8 (13%) | 5 (16%) | 3 (9%) |
| Father/mother | 44 (70%) | 20 (65%) | 24 (75%) |
| Other | 11 (17%) | 6 (19%) | 5 (16%) |
| Time Caring: n (months %) |  |  |  |
| <12 | 10 (16%) | 6 (19%) | 4 (12%) |
| 12-48 | 28 (44%) | 13 (42%) | 15 (47%) |
| 48+ | 25 (40%) | 12 (39%) | 13 (41%) |
| Time week: n (days %) |  |  |  |
| 1-2 | 3 (5%) | 1 (3%) | 2 (6%) |
| 3-4 | 6 (9%) | 3 (10%) | 3 (9%) |
| 5-7 | 54 (86%) | 27 (87%) | 27 (85%) |
| Time day: n (hours %) |  |  |  |
| 3< | 9 (14%) | 5 (16%) | 4 (13%) |
| 3-7 | 24 (38%) | 14 (45%) | 10 (31%) |
| 8-11 | 7 (11%) | 5 (16%) | 2 (6%) |
| 12+ | 23 (37%) | 7 (23%) | 16 (50%) |
| Residing with care receiver: n (yes %) | 49 (78%) | 24 (78%) | 25 (78%) |
| Individual is the only caregiver: n (yes %) | 31 (50%) | 18 (58%) | 13 (41%) |
| Highest education level: n (%) |  |  |  |
| High school or lower | 4 (6%) | 3 (10%) | 1 (3%) |
| Professional/vocational training | 17 (27%) | 9 (29%) | 8 (25%) |
| College or applied science education | 6 (10%) | 2 (6%) | 4 (12%) |
| University degree | 36 (57%) | 17 (55%) | 19 (60%) |
| Marital status: n (%) |  |  |  |
| Single | 12 (19%) | 7 (23%) | 5 (16%) |
| Married/partner | 39 (62%) | 18 (58%) | 21 (65%) |
| Divorced/widowed or other | 12 (19%) | 6 (19%) | 6 (19%) |

^1^Biliunaite I, Kazlauskas E, Sanderman R, Truskauskaite-Kuneviciene I, Dumarkaite A, Andersson G. Internet-Based Cognitive Behavioral Therapy for Informal Caregivers: Randomized Controlled Pilot Trial. Journal of Medical Internet Research. 2021;23(4):e21466.
